# A frequent variant in the human bile salt export pump gene *ABCB11* is associated with hepatitis C virus infection, but not liver stiffness in a German population

**DOI:** 10.1186/1471-230X-12-63

**Published:** 2012-06-08

**Authors:** Roman Müllenbach, Susanne N Weber, Marcin Krawczyk, Vincent Zimmer, Christoph Sarrazin, Frank Lammert, Frank Grünhage

**Affiliations:** 1 Department of Medicine II, Saarland University Medical Center, Homburg, Germany; 2 Department of Medicine I, Goethe University Hospital, Frankfurt, Germany

## Abstract

**Background:**

The human ATP-binding cassette, subfamily B, member 11 (*ABCB11)* gene encodes the bile salt export pump, which is exclusively expressed at the canalicular membrane of hepatocytes. A frequent variant in the coding region, c.1331 T > C, leading to the amino acid exchange p.V444A, has been associated with altered serum bile salt levels in healthy individuals and predisposes homozygous carriers of the [C] allele for obstetric cholestasis. Recently, elevated bile salt levels were shown to be significantly associated with rates and risk of cirrhosis in patients with chronic hepatitis C virus (HCV) infection treated with pegylated interferon-α2 and ribavirin, suggesting a potential role for bile salt levels in HCV treatment outcomes and in the fibrogenic evolution of HCV-related liver disease. The **aim** of this study was to investigate a possible association of *ABCB11* c.1331 T > C with hepatitis C virus (HCV) infection and fibrosis stages as assessed by non-invasive transient elastography in a German cohort of patients.

**Methods:**

*ABCB11* c.1331 T > C genotype was determined by allelic discrimination assay in 649 HCV infected cases and 413 controls. Overall, 444 cases were staged for fibrotic progression by measurement of liver stiffness.

**Results:**

Homo- or heterozygous presence of the frequent [C] allele was associated with HCV positivity (OR = 1.41, CI = 1.02 - 1.95, p = 0.037). No association was detectable between the *ABCB11* c.1331 T > C genotype and increased liver stiffness.

**Conclusions:**

Our data confirm that homozygous presence of the major [C] allele of *ABCB11* c.1331 T > C is a genetic susceptibility factor for HCV infection, but not for liver fibrosis.

## Background

Hepatitis C virus (HCV) is one of the most common causes of fibrotic liver disease. It is estimated that there are 5 million carriers in Europe, and up to 500.000 chronically HCV-infected people in Germany [1]. These individuals are at high risk of developing progressive liver disease, including cirrhosis and hepatocellular carcinoma [2,3]. Genetic factors influence susceptibility to HCV infection but also progression of the disease. Due to the viral life cycle and its reliance on the infected cell, host genetic factors are likely to exert a strong influence on these processes. This has been shown for example in a study compromising Irish women infected with an HCV-contaminated anti-D immunoglobulin acquired from the same acutely infected person [4,5]. The analysis of liver samples acquired in average almost two decades after the infection showed a highly variable disease progression, which was apparently not affected by the type of the hepatitis C virus itself but by the host genetic background and lifestyle [4,5]. A number of genetic studies have reported associations of variants in candidate genes such as tumor necrosis factor [6-8], interleukin 10 [9], HLA subtypes [10-12] and CC chemokine receptor 5 [13] with susceptibility and progression of HCV infection. Huang et al. [14] used a machine learning approach and two independent Caucasian cohorts to identify a seven-gene signature that allows better prognosis of fibrosis progression in HCV infected individuals than established clinical parameters. This study and the follow-up by Marcolongo et al. in an Italian population [15] illustrate the feasibility of genetic risk assessment for cirrhosis prediction. They also accentuate the need to identify additional genetic factors that contribute towards HCV susceptibility and subsequent fibrotic progression. A comprehensive map of cirrhosis-associated variants might permit verification of interacting and modifying loci in selected patient groups, which would in turn improve the predictive values of polygenic cirrhosis risk scores.

Previously a common, non-conservative polymorphism c.1331 T > C (p.V444A) in the hepatobiliary bile salt transporter *ABCB11* (also known as bile salt export pump, *BSEP*) was identified as a risk factor for cholestatic liver diseases, in particular drug-induced cholestasis [16] and intrahepatic cholestasis of pregnancy [17,18]. The human bile salt export pump represents the central canalicular transporter of bile salts from hepatocytes into the bile canaliculus [19,20]. In normal conditions, ABCB11 together with other hepatobiliary proteins (i.e. ABCB4 and ABCG5/8 transporting phospholipids and sterols into bile, respectively) maintain proper concentrations of biliary constituents. This, in turn, preserves bile flow and minimises the toxic properties of biliary components. Interestingly, the analysis of hepatobiliary transporter expression has demonstrated that the functional effect of the p.A444V polymorphism is due to markedly lower expression of ABCB11 [21,22] rather than impaired function of the protein [16].

Lately two reports have underscored an association between the c.1331 T > C variant and both response to antiviral treatment [23] and the presence of cirrhosis in patients with chronic HCV infection [24]. The analysis of 151 individuals treated with ribavirin and pegylated interferon demonstrated that those carrying the genotype [TT] and infected with HCV genotypes 2 or 3 show increased sustained virological response (SVR) rates as compared to [CC] individuals [23]. Additionally, the [CC] genotype has been reported to increase the risk of developing liver cirrhosis in chronic HCV infection [24].

To validate and extend previous results concerning possible associations of the common polymorphism c.1331 T > C in the bile salt export pump with HCV infection and fibrosis progression, we analysed its genotype distribution in a larger cohort of 649 patients with chronic HCV infection, in 444 of whom liver fibrosis was staged non-invasively by transient liver elastography.

## Methods

### Patients

Overall, we included 649 patients with chronic HCV infection. The cohort consisted of two ethnically matched subgroups of patients: 487 patients with active chronic HCV infection were prospectively recruited at the Department of Medicine I of the University Hospital Bonn. Patients were included in the study if they were positive for anti-HCV antibodies and had detectable HCV-RNA in serum. Patients with other viral infections (HBV, HDV, HIV) or chronic liver diseases were excluded. Baseline clinical characteristics of the studied cohort have been published previously [25] and are summarised in Table 1. In addition, we included 162 treatment-naïve HCV patients recruited at the Department of Medicine II of the Saarland University Hospital in Homburg. However, since there were incomplete phenotyping data in terms of liver stiffness measurements, this group of patients was included only in the analysis of HCV-positive versus HCV-negative individuals. Patients without TE were also eligible for the study when they presented with overt signs of liver cirrhosis such as ascites, signs of portal hypertension, or coagulopathy. In addition, 413 HCV-negative individuals were recruited from the outpatient clinic of the Department of Medicine I of the University Hospital Bonn. The most frequent aetiologies of liver damage were alcohol (97), non-alcoholic fatty liver disease (63), chronic hepatitis B virus infection (73), autoimmune hepatitis (29), haemochromatosis (26), primary biliary cirrhosis (23), and primary sclerosing cholangitis (13). The median age of the HCV-negative individuals was 53 years (range 18-88), and the gender ratio was 1,4:1. All patients gave written informed consent. The study was approved by independent ethics committees at the Universities of Bonn and Saarland.

**Table 1:** Patient cohort characteristics

**Factor**	**Bonn cohort**	**Homburg cohort**
	**(n = 487)**	**(n = 162)**
Age (years)	48 (19-83)	49 (21-79)
Gender		
Male	315 (64.7%)	75 (46.3%)
Female	172 (35.3%)	87 (53.7%)
BMI (kg/m^2^)	24.2 (14.8 - 39.8)	25.7 (17.0 - 43.0)
TE (kPa)	7.1 (2.7 - 75.0)	n.a.
HCV genotypes		
HCV 1	170 (46%)	139 (86%)
HCV non-1	199 (54%)	23 (14%)
unknown	118	

Continuous variables are presented as medians and ranges.

Abbreviations: BMI, body mass index; n. a., not available; TE, transient elastography.

### Assessment of liver fibrosis with transient elastography

In total, 444 patients with chronic HCV infection were examined by transient elastography (TE). Although liver biopsy represents a “gold standard” of quantifying liver status in patients with chronic liver diseases, we have lately shown that TE is a feasible phenotyping method in genetic studies aiming at identification of novel variants affecting liver fibrogenesis [25]. Hence, in our current study we determined liver stiffness by TE. In brief, the tip of the probe transducer was placed on the skin between the rib bones at the level of the right lobe of the liver. The measurement depth ranged between 25 and 65 mm below the skin surface. In case of ascites, elastography was preceded by large-volume paracentesis. Ten measurements were performed in each individual, and subsequently the median value was taken as representative. Patients were assigned to different fibrosis stages according to TE results, using previously published cut-off values; in particular, patients with TE results ≥ 13.0 kPa or overt clinical signs were diagnosed to suffer from liver cirrhosis, since TE cut-offs in this range were demonstrated to be optimal for the diagnosis of cirrhosis [26].

### Genotyping

Genomic DNA was isolated from EDTA anti-coagulated blood using the membrane based QIAamp DNA extraction protocol (Qiagen, Hilden, Germany). DNA concentrations were determined fluorometrically (Bio-Rad Laboratories, Hercules, CA, USA), employing the dye PicoGreen (Molecular Probes, Leiden, Netherlands).

The *ABCB11* polymorphism c.1331 T > C (rs2287622) [17,22-24] was genotyped using solution-phase hybridization reactions with 5'-nuclease and fluorescence detection (TaqMan assays) on the 7300 Real-Time PCR System (Applera, Norwalk, CT, USA). PCR reactions contained 20 ng genomic DNA, 1× TaqMan Universal Master Mix, 900 nM of each primer, and 200 nM of VIC-labeled and FAM-labeled probes in 25 μl-reactions. Amplification conditions were 95°C for 10 min, followed by 40 cycles at 92°C for 15 s, and 60°C for 1 min. Primers and probes are available from the Applera database (http://www.appliedbiosystems.com/).

### Statistics

Data are given as means ± SD, unless stated otherwise. Kolmogorov-Smirnov’s test was used to determine whether data had a normal distribution.

Exact tests were performed to ensure consistency of the genotyping results with Hardy-Weinberg equilibrium (HWE) (http://ihg2.helmholtz-muenchen.de/cgi-bin/hw/hwa1.pl). Association case–control analysis was performed to investigate the role of the *ABCB11* variant in the susceptibility to HCV infection. Logistic regression analysis for the presence of cirrhosis was performed with SPSS 18.0 (SPSS, Munich, Germany) and DATADESK (Data Description, Ithaca, NY, USA). To compensate for multiple testing (5 parameters), a P-value below 0.018 was considered significant in regression analysis.

## Results

### Carriers of the *ABCB11* c.1331 C allele are at increased risk of HCV infection

Overall, 649 patients with active chronic HCV infection and 413 control individuals were genotyped. We did not observe a significant difference in allele frequencies between patients and controls (P = 0.06). Interestingly, genotype analysis presented in Table 2 demonstrates that homo- and heterozygous carriers of the risk allele [C] were 1.4 times as likely to suffer from active HCV infection than patients with genotype [TT] (P = 0.037).

**Table 2:** **Genotyping results for*ABCB11 *c.1331 T > C from patients and controls**

***ABCB11*p.V444A (c.1331 T > C) ** **alleles/genotypes**	**Counts (frequencies) of alleles/genotypes**	
	**Cases (N = 649)**	**Controls (N = 413)**
[T]	511 (0.39)	359 (0.43)
[C]	787 (0.61)	467 (0.57)
[TT]	97 (0.15)	82 (0.20)
[TC]	317 (0.49)	195 (0.47)
[CC]	235 (0.36)	136 (0.33)
**Tests for association**	**chi**^**2**^	**P**
Allele frequency difference	3.50	0.061
Armitage’s trend test	3.49	0.062
**OR statistics**	**OR (95% CI)**	**P**
[C] ↔ [T]	1.18 (0.99 - 1.41)	0.061
[CC] ↔ [TT]	1.46 (1.02 - 2.10)	0.040
[CC + CT] ↔ [TT]	1.41 (1.02 - 1.95)	0.037

Patients with HCV infections are regarded as cases.

Abbreviations: *A*, alanine; *ABCB11*, ATP-binding cassette, subfamily B, member 11; *CI*, confidence interval; *OR*, odds ratio; *p*, protein (amino acid number), *V*, valine.

### *ABCB11* c.1331 T > C variant does not increase the risk of liver fibrosis or cirrhosis in chronic hepatitis C patients

To assess a potential impact of the *ABCB11* c.1331 risk genotype [CC] on cirrhosis, we performed univariate and multivariate logistic regression analyses. No significant correlation was detected between the presence of cirrhosis (TE ≥ 13kPa) and the presence of the [CC] genotype in patients (P = 0.925). The only factor significantly associated with the presence of cirrhosis in multivariate analysis was the age of the patients (P = 0.006) (Table 3).

**Table 3:** Univariate and multivariate analysis of the presence of cirrhosis (TE ≥ 13kPa)

**Univariate analysis:**			
Parameter	OR	95% CI	P value
Age (>40 vs ≤40 years)	2.405	1.311 - 4.411	0.005
Gender (male vs. female)	1.466	0.914 - 2.350	0.131
HCV genotype (1 vs 2/3)	1.263	0. 771-2.070	0.353
BMI (>25 vs ≤25 kg/m^2^)	1.676	1.038 - 2.707	0.034
*ABCB11* (CC vs CT + TT)	1.022	0.650 - 1.608	0.925
**Multivariate analysis:**			
Parameter	OR	95% CI	P value
Age (>40 vs ≤40 years)	2.714	1.341 - 5.496	0.006
BMI (>25 vs ≤25 kg/m^2^)	1.622	1.000 - 2.632	0.050

**Figure 1:**
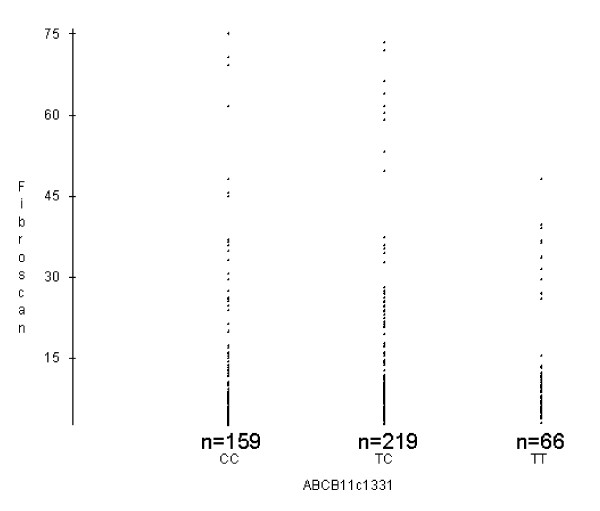
**Scatter plot of TE results [kPa] for*ABCB11*c.1331 T > C genotypes (n = 444).**

Figure 1 illustrates that scatterplot analysis is indicative of a higher frequency of individuals with more severe cirrhosis, i.e. TE levels ≥ 45 kPa among carriers of genotypes [CC] or [CT] as compared to homozygous [TT] individuals. This observation does not reach statistical significance in numerical comparison due to the low numbers of homozygous [TT] carriers.

## Discussion

The present study shows that a common variant in the human bile salt export pump *ABCB11* is overrepresented in patients with HCV infection. The reason underlying this observation is speculative, although it might be caused by differences in spontaneous recovery from infection and sustained virological response (SVR) rates, as was observed in patients bearing HCV genotypes 2 and 3 in a previous study: Iwata et al. [23] were able to show that the effect might be mediated by serum bile salt concentrations, with low levels (< 8 μM) being associated with SVR in their cohort. This is not the first time that bile salts or genes involved in their metabolism have been implicated in the pathogenesis of HCV infection. Ursodeoxycholic acid has been previously proven in various trials to be beneficial in patients suffering from liver damage due to chronic infection, albeit not via modulation of SVR[27-29]. Contrastingly, increased serum levels of bile salts are associated with negative outcomes in HCV therapy [30]. The observed association between a polymorphism that impinges bile salt transport from the hepatocyte into the canaliculus offers an explanation of this conundrum: Viral replication might be boosted inside the hepatocyte by relatively high bile salt concentrations [30]. A variant of the bile salt export pump with lower expression level resulting in reduced transport activity, such as the common [C] allele of c.1331 T > C, might lead to transient increases in hepatocellular bile salt concentration, depending on the efficiency of the sinusoidal import.

Ursodeoxycholic acid as rather weak FXR agonist is known to increase the expression and activity of bile salt transporters by transcriptional as well as multiple post-transcriptional mechanisms and to contribute to SHP-mediated inhibition of bile salt synthesis [31,32]. *In vitro* experiments using HCV replicon-harboring cells have shown that the impact of bile salts on HCV replication is mediated by the action of FXR rather than via direct effects. FXR antagonisation by guggulsterone blocked the bile salt-induced up-regulation of virus replication; furthermore, guggulsterone inhibited basal levels of HCV replication [30,33]. Hence, it is possible that HCV "hijacks" or uses transcriptional activation via FXR. Whatever the mode of action by which HCV takes advantage of FXR signalling, it appears to be advantageous for the hepatotropic virus to use central functions of hepatocytes for its own end.

HCV replication is tightly linked to lipid metabolism, and bile salts are crucially involved in fat absorption and transport. This reinforces the key roles of bile salt responsive regulatory molecules such as FXR and TGR5 as potential host factors modulating HCV replication, not necessarily through direct effect of bile salts but via feedback regulation of lipid metabolism. In infected cells, HCV core protein accumulates on the surface of lipid droplets [34,35]. Other functional proteins, such as RNA binding replicase NS5A, are targeted to lipid droplets, and this targeting appears to be crucial for HCV assembly [36]. A recent combined transcriptome and proteome analysis revealed the impact of HCV infection on previously unknown metabolic pathways, particularly an increase in cellular cholesterol and free fatty acid levels [37]. One possible reason for this "metabolic reprogramming" might be the dependency of HCV replication on lipid metabolism as outlined above. Considering the central role of ABCB11 in bile flow, it is not inconceivable to conclude that common variants of this transporter are associated with variations in plasma cholesterol levels. In fact, a meta-analysis of genome-wide association scans supports this reasoning [38], thus providing further evidence for another potential connection between bile salt transport and HCV replication. The results from our study are indicative of a role for the major allele of the *ABCB11* c.1331 variant in conveying susceptibility to persistent HCV infection, enhancing this link.

Iwata and colleagues observed an overrepresentation of the [C] allele at *ABCB11 *c.1331 in HCV patients (n = 206) compared with controls (n = 110) in the Swiss Hepatitits C cohort (allelic frequency = 62.9%), although no significant difference in median bile salt serum concentration could be detected between bearers of homozygous [C] or [T] alleles [24]. However, a comparison of fibrosis scores from 178 patients revealed a skewed distribution: Carriers of homozygous [C] allele were overrepresented among cirrhotic patients, and the [CC] genotype was an independent risk factor for cirrhosis in multivariate analysis (p = 0.010).

Our investigations into a connection between *ABCB11* c.1331 T > C and liver stiffness as measured by TE (Fibroscan) did not reveal any significant associations. This may be due to methodolocial differences in assessing fibrosis/cirrhosis [25,26]. Scatterplot analysis of TE results from the different genotypes seems to indicate a prevalence of lower values in patients bearing the [TT] genotype (Figure 1), which would be in agreement with the data from the Swiss cohort, but the numbers of homozygous carriers of the minor allele in our cohort were too low to reach statistical significance.

Cirrhosis was significantly associated with the age of patients (p = 0.006), which may reflect longer duration of chronic HCV infection. However, since the exact time point of infection was unknown in the majority of our patients, we used age as surrogate marker in our statistical analyses.

Interestingly, when comparing allelic distribution in our cohort with figures from the Swiss Hepatitis C cohort [24], *ABCB11* c.1331 T > C is equally skewed towards an overrepresentation of the [C] allele among British patients with non-alcoholic fatty liver disease in the study by Iwata et al., pointing towards a potential role of ABCB11 via its impact on lipid metabolism rather than viral replication. The development of diet-induced obesity and hypercholesterolemia following hepatocye-specific overexpression of *ABCB11* in mice is a pointer in the same direction [39].

Based on the assumption that populations from both the Swiss study and our cohort are from a similar ethnic background, we speculate that the major [C] allele of *ABCB11* c.1331 T > C might be a high-frequency low-risk contributor towards susceptibility for various complex liver diseases triggered by external stimuli and involving lipid metabolism [40].

## Conclusions

Individuals bearing the homozygous [C] allele at *ABCB11* c.1331 T > C are overrepresented in a German cohort of HCV patients. This variant has been linked to lower expression of the resulting ABCB11 protein, which supports a role of bile salts in the hepatocytes' response to HCV infection.

## Competing interests

The authors declare that they have no competing interests.

## Authors’ contributions

RM: Statistical evaluation, manuscript conception and writing. SW: Experimental conception and supervision. MK: Liver stiffness assessment, statistical evaluation, manuscript writing. VZ: Patient recruitment, manuscript writing. CS: Patient recruitment. FL: Experimental conception, manuscript writing. FG: Patient recruitment and liver stiffness assessment, statistical evaluation, manuscript conception. All authors read and approved the final manuscript.

## Pre-publication history

The pre-publication history for this paper can be accessed here:

http://www.biomedcentral.com/1471-230X/12/63/prepub
